# Trilithium scandium bis­(orthoborate)

**DOI:** 10.1107/S1600536808014797

**Published:** 2008-05-21

**Authors:** Lizhong Mao, Tianyong Zhou, Ning Ye

**Affiliations:** a Fujian Institute of Research on the Structure of Matter, Chinese Academy of Sciences, Fuzhou, Fujian 350002, People’s Republic of China

## Abstract

Single crystals of the title compound, Li_3_Sc(BO_3_)_2_, have been obtained by spontaneous nucleation from a high-temperature melt. The title compound adopts a framework structure and is composed of distorted [ScO_6_] octa­hedra, [LiO_4_] tetra­hedra, [LiO_4_] recta­ngles and isolated [BO_3_] triangles. Except for the Sc and one Li atom (both on inversion centres), all atoms are in general positions.

## Related literature

For a review of structural data of BO_3_ groups, see: Zobetz (1982 ▶). For sodium scandium borates, see: Becker & Held (2001 ▶); Zhang *et al*. (2006 ▶).

## Experimental

### 

#### Crystal data

Li_3_Sc(BO_3_)_2_
*M* *_r_* = 183.4
Monoclinic, 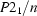

*a* = 4.7831 (17) Å
*b* = 5.954 (2) Å
*c* = 8.163 (3) Å
β = 90.702 (9)°
*V* = 232.44 (15) Å^3^
*Z* = 2
Mo *K*α radiation
μ = 1.53 mm^−1^
*T* = 293 (2) K
0.12 × 0.10 × 0.10 mm

#### Data collection

Rigaku Mercury CCD diffractometer
Absorption correction: multi-scan (*CrystalClear*; Rigaku, 2000 ▶) *T*_min_ = 0.833, *T*_max_ = 0.858
1734 measured reflections
534 independent reflections
518 reflections with *I* > 2σ(*I*)
*R*_int_ = 0.015

#### Refinement

*R*[*F* ^2^ > 2σ(*F* ^2^)] = 0.017
*wR*(*F* ^2^) = 0.058
*S* = 1.10
534 reflections
58 parameters
Δρ_max_ = 0.28 e Å^−3^
Δρ_min_ = −0.23 e Å^−3^

### 

Data collection: *CrystalClear* (Rigaku, 2000 ▶); cell refinement: *CrystalClear*; data reduction: *CrystalClear*; program(s) used to solve structure: *SHELXS97* (Sheldrick, 2008 ▶); program(s) used to refine structure: *SHELXL97* (Sheldrick, 2008 ▶); molecular graphics: *DIAMOND* (Brandenburg, 2004 ▶); software used to prepare material for publication: *enCIFer* (Allen *et al.*, 2004 ▶).

## Supplementary Material

Crystal structure: contains datablocks global, I. DOI: 10.1107/S1600536808014797/wm2180sup1.cif

Structure factors: contains datablocks I. DOI: 10.1107/S1600536808014797/wm2180Isup2.hkl

Additional supplementary materials:  crystallographic information; 3D view; checkCIF report

## Figures and Tables

**Table:** 

Sc—O1	2.0854 (12)
Sc—O2^i^	2.1101 (12)
Sc—O3^i^	2.1197 (13)
Li1—O2	2.0107 (12)
Li1—O1^ii^	2.1173 (12)
Li2—O1^iii^	1.896 (3)
Li2—O2	1.946 (3)
Li2—O3^iv^	1.983 (3)
Li2—O3^i^	2.137 (3)
B—O2	1.376 (2)
B—O3^v^	1.384 (2)
B—O1^vi^	1.385 (2)

**Table:** 

O2—B—O3^v^	122.09 (14)
O2—B—O1^vi^	119.13 (14)
O3^v^—B—O1^vi^	118.72 (14)

Symmetry codes: (i) 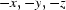; (ii) 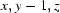; (iii) 
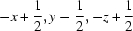
; (iv) 
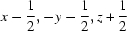
; (v) 
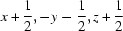
; (vi) 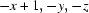.

**Fig. 1.**
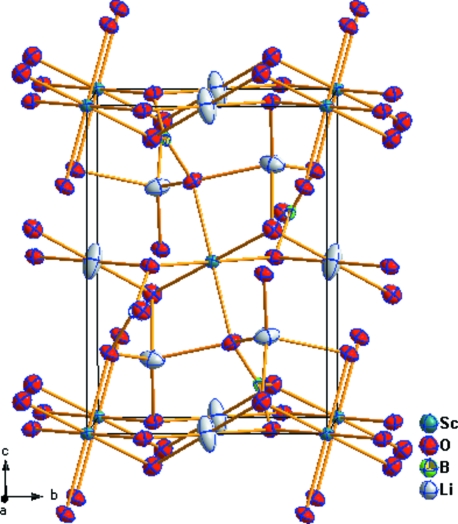
The structure of (I) in a projection approximatly along the [001] direction with displacement ellipsoids drawn at the 85% probability level.

**Fig. 2.**
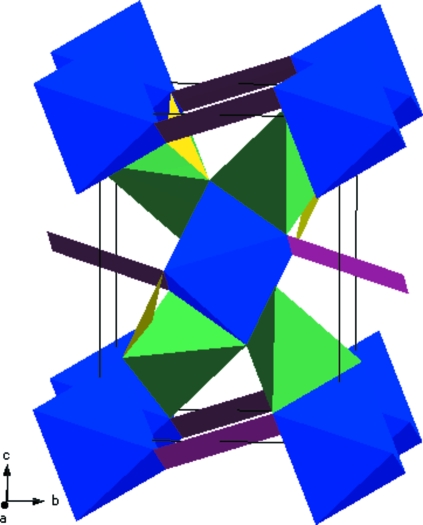
The structure of (I) given in the polyhedral description. [ScO6] octahedra are blue, [LiO4] tetrahedra are green, [LiO4] rectangles are purple, and [BO3] units are yellow.

## supplementary crystallographic information

### Comment

Li_3_Sc(BO_3_)_2_, (I), was found from analysis of phase equilibria in the system Li_2_O—Sc_2_O_3_—B_2_O_3_, in which it is the first characterized pseudo-ternary phase. For the heavier Na homologue, two phases are already known, viz. Na_3_Sc_2_(BO_3_)_3_ (Zhang *et al.*, 2006) and NaScB_2_O_5_ (Becker & Held, 2001).

The framework structure of (I) is made up of distorted [ScO_6_] octahedra, [LiO_4_] tetrahedra, [LiO_4_] rectangles and [BO_3_] triangles as single building units. The [ScO_6_] octahedra are linked via [LiO_4_] rectangles by sharing edges to form columns parallel to [010]. The columns are linked to each other through [LiO_4_] tetrahedra and [BO_3_] triangles by sharing edges and corners (Figs 1 and 2).

The B atom is coordinated to three oxygen atoms forming nearly trigonal planar [BO_3_]^3-^ anions. The B—O bond lengths range from 1.376 (2) to 1.385 (2) Å, and the O—B—O angles are close to 120° (Table 1), values that are typical for BO_3_ groups (Zobetz, 1982). The Sc^3+^ cation is coordinated by six oxygen atoms to form a distorted [ScO_6_] ocahedron with Sc—O bond lengths ranging from 2.0854 (12) to 2.1197 (13) Å. There are two crystallographically different Li atoms. One is situated on an inversion centre (1 symmetry) and is coordinated to four oxygen atoms forming a nearly planar [LiO_4_] rectangle with Li1—O bond lengths ranging from 2.0107 (12) to 2.1173 (12) Å. The other Li atom is also coordinated to four O atoms, but is in the centre of a distorted tetrahedron with Li2—O bond lengths from 1.896 (3) to 2.137 (3) Å (Table 1). The average Li—O bond length of the [Li1O_4_] rectangle (2.064 Å) is slightly longer than that of the [Li2O_4_] tetrahedron (1.991 Å).

### Experimental

Single crystals of compound (I) were grown using a LiBO_2_-containing flux. The composition of the mixture for crystal growth was 4:1:4 of Li_2_CO_3_ (Sinopharm Reagents, 99.99%), Sc_2_O_3_ (Sinopharm Reagents, 4 N), and B_2_O_3_ (Sinopharm Reagents, 99%). This mixture was heated in a platinum crucible to 1373 K, held at this temperature for several hours, and then cooled at a rate of 10 K/h from 1373 to 873 K. The remaining flux attached to the crystals was readily dissolved in water. Crystals with an average size of 0.5 mm and mostly block shaped habit were obtained.

### Crystal data

**Table:**

Li_3_Sc(BO_3_)_2_	*F*_000_ = 176
*M**_r_* = 183.4	*D*_x_ = 2.62 Mg m^−^^3^
Monoclinic, *P*2_1_/*n*	Mo *K*α radiation λ = 0.71073 Å
Hall symbol: -P 2yn	Cell parameters from 623 reflections
*a* = 4.7831 (17) Å	θ = 4.2–23.6º
*b* = 5.954 (2) Å	µ = 1.53 mm^−^^1^
*c* = 8.163 (3) Å	*T* = 293 (2) K
β = 90.702 (9)º	Block, colourless
*V* = 232.44 (15) Å^3^	0.12 × 0.10 × 0.10 mm
*Z* = 2	

### Data collection

**Table:**

Rigaku Mercury CCD diffractometer	534 independent reflections
Radiation source: Sealed Tube	518 reflections with *I* > 2σ(*I*)
Monochromator: Graphite Monochromator	*R*_int_ = 0.015
Detector resolution: 14.6306 pixels mm^-1^	θ_max_ = 27.5º
*T* = 293(1) K	θ_min_ = 4.2º
CCD_Profile_fitting scans	*h* = −6→4
Absorption correction: multi-scan(CrystalClear; Rigaku, 2000)	*k* = −7→7
*T*_min_ = 0.833, *T*_max_ = 0.858	*l* = −10→10
1734 measured reflections	

### Refinement

**Table:**

Refinement on *F*^2^	Primary atom site location: structure-invariant direct methods
Least-squares matrix: full	*w* = 1/[σ^2^(*F*_o_^2^) + (0.0318*P*)^2^ + 0.216*P*] where *P* = (*F*_o_^2^ + 2*F*_c_^2^)/3
*R*[*F*^2^ > 2σ(*F*^2^)] = 0.017	(Δ/σ)_max_ < 0.001
*wR*(*F*^2^) = 0.058	Δρ_max_ = 0.28 e Å^−^^3^
*S* = 1.10	Δρ_min_ = −0.23 e Å^−^^3^
534 reflections	Extinction correction: SHELXL97 (Sheldrick, 2008)
58 parameters	Extinction coefficient: ?

### Special details

**Table:**

Geometry. All e.s.d.'s (except the e.s.d. in the dihedral angle between two l.s. planes) are estimated using the full covariance matrix. The cell e.s.d.'s are taken into account individually in the estimation of e.s.d.'s in distances, angles and torsion angles; correlations between e.s.d.'s in cell parameters are only used when they are defined by crystal symmetry. An approximate (isotropic) treatment of cell e.s.d.'s is used for estimating e.s.d.'s involving l.s. planes.

### Fractional atomic coordinates and isotropic or equivalent isotropic displacement parameters (Å^2^)

**Table:**

	*x*	*y*	*z*	*U*_iso_*/*U*_eq_	
Sc	0	0	0	0.00510 (15)	
Li1	0	−0.5	0	0.0209 (10)	
Li2	−0.0144 (6)	−0.2513 (5)	0.2977 (4)	0.0133 (6)	
B	0.5149 (4)	−0.3045 (3)	0.1254 (2)	0.0061 (3)	
O1	0.3101 (2)	0.24622 (18)	0.00179 (14)	0.0077 (2)	
O2	0.2330 (2)	−0.26155 (19)	0.11029 (14)	0.0086 (3)	
O3	0.1280 (2)	−0.08686 (19)	−0.23947 (13)	0.0086 (3)	

### Atomic displacement parameters (Å^2^)

**Table:**

	*U*^11^	*U*^22^	*U*^33^	*U*^12^	*U*^13^	*U*^23^
Sc	0.0050 (2)	0.0054 (2)	0.0049 (2)	−0.00002 (13)	0.00011 (14)	0.00009 (12)
Li1	0.015 (2)	0.012 (2)	0.035 (3)	0.0000 (15)	−0.008 (2)	−0.0062 (17)
Li2	0.0119 (13)	0.0182 (15)	0.0098 (13)	−0.0025 (11)	−0.0018 (10)	0.0015 (10)
B	0.0074 (7)	0.0042 (7)	0.0067 (7)	−0.0009 (6)	0.0000 (6)	−0.0014 (6)
O1	0.0065 (5)	0.0095 (5)	0.0070 (5)	−0.0013 (4)	0.0004 (4)	0.0005 (4)
O2	0.0060 (5)	0.0099 (5)	0.0099 (6)	0.0013 (4)	0.0000 (4)	0.0012 (4)
O3	0.0084 (5)	0.0102 (6)	0.0071 (5)	0.0006 (4)	−0.0009 (4)	−0.0023 (4)

### Geometric parameters (Å, °)

**Table:**

Sc—O1	2.0854 (12)	Li2—O1^x^	1.896 (3)
Sc—O1^i^	2.0854 (12)	Li2—O2	1.946 (3)
Sc—O2^i^	2.1101 (12)	Li2—O3^xi^	1.983 (3)
Sc—O2	2.1101 (12)	Li2—O3^i^	2.137 (3)
Sc—O3^i^	2.1197 (13)	Li2—B^viii^	2.659 (3)
Sc—O3	2.1197 (13)	Li2—B^xi^	2.697 (3)
Sc—Li2^i^	2.855 (3)	Li2—B^xii^	2.733 (4)
Sc—Li2	2.855 (3)	Li2—Sc^ix^	3.226 (3)
Sc—Li1^ii^	2.9768 (11)	Li2—Li1^iii^	3.234 (3)
Sc—Li1	2.9768 (11)	Li2—Sc^x^	3.297 (3)
Sc—Li2^iii^	3.226 (3)	B—O2	1.376 (2)
Sc—Li2^iv^	3.226 (3)	B—O3^xiii^	1.384 (2)
Li1—O2	2.0107 (12)	B—O1^xiv^	1.385 (2)
Li1—O2^v^	2.0107 (12)	B—Li2^xv^	2.659 (3)
Li1—O1^vi^	2.1173 (12)	B—Li2^iv^	2.697 (3)
Li1—O1^i^	2.1173 (12)	B—Li2^x^	2.733 (4)
Li1—B^vii^	2.8008 (18)	B—Li1^xv^	2.8008 (18)
Li1—B^viii^	2.8008 (18)	O1—B^xiv^	1.385 (2)
Li1—Li2^v^	2.847 (3)	O1—Li2^xii^	1.896 (3)
Li1—Li2	2.847 (3)	O1—Li1^ii^	2.1173 (12)
Li1—Sc^vi^	2.9768 (11)	O3—B^xvi^	1.384 (2)
Li1—Li2^ix^	3.234 (3)	O3—Li2^iv^	1.983 (3)
Li1—Li2^iv^	3.234 (3)	O3—Li2^i^	2.137 (3)
			
O1—Sc—O1^i^	180.00 (4)	O1^x^—Li2—O3^i^	109.04 (14)
O1—Sc—O2^i^	81.73 (5)	O2—Li2—O3^i^	90.58 (12)
O1^i^—Sc—O2^i^	98.27 (5)	O3^xi^—Li2—O3^i^	101.95 (13)
O1—Sc—O2	98.27 (5)	O2—B—O3^xiii^	122.09 (14)
O1^i^—Sc—O2	81.73 (5)	O2—B—O1^xiv^	119.13 (14)
O2^i^—Sc—O2	180.00 (8)	O3^xiii^—B—O1^xiv^	118.72 (14)
O1—Sc—O3^i^	92.04 (4)	B^xiv^—O1—Li2^xii^	109.58 (13)
O1^i^—Sc—O3^i^	87.96 (4)	B^xiv^—O1—Sc	127.37 (10)
O2^i^—Sc—O3^i^	93.23 (5)	Li2^xii^—O1—Sc	111.74 (11)
O2—Sc—O3^i^	86.77 (5)	B^xiv^—O1—Li1^ii^	104.23 (9)
O1—Sc—O3	87.96 (4)	Li2^xii^—O1—Li1^ii^	110.73 (10)
O1^i^—Sc—O3	92.04 (4)	Sc—O1—Li1^ii^	90.19 (5)
O2^i^—Sc—O3	86.77 (5)	B—O2—Li2	122.67 (13)
O2—Sc—O3	93.23 (5)	B—O2—Li1	116.47 (10)
O3^i^—Sc—O3	180.00 (5)	Li2—O2—Li1	92.02 (10)
O2—Li1—O2^v^	180.00 (6)	B—O2—Sc	133.37 (10)
O2—Li1—O1^vi^	96.68 (5)	Li2—O2—Sc	89.39 (10)
O2^v^—Li1—O1^vi^	83.32 (5)	Li1—O2—Sc	92.47 (5)
O2—Li1—O1^i^	83.32 (5)	B^xvi^—O3—Li2^iv^	102.89 (13)
O2^v^—Li1—O1^i^	96.68 (5)	B^xvi^—O3—Sc	137.30 (10)
O1^vi^—Li1—O1^i^	180	Li2^iv^—O3—Sc	103.64 (10)
O1^x^—Li2—O2	111.51 (16)	B^xvi^—O3—Li2^i^	99.62 (12)
O1^x^—Li2—O3^xi^	124.23 (16)	Li2^iv^—O3—Li2^i^	135.08 (11)
O2—Li2—O3^xi^	113.33 (15)	Sc—O3—Li2^i^	84.24 (9)

Symmetry codes: (i) −*x*, −*y*, −*z*; (ii) *x*, *y*+1, *z*; (iii) −*x*−1/2, *y*+1/2, −*z*+1/2; (iv) *x*+1/2, −*y*−1/2, *z*−1/2; (v) −*x*, −*y*−1, −*z*; (vi) *x*, *y*−1, *z*; (vii) −*x*+1, −*y*−1, −*z*; (viii) *x*−1, *y*, *z*; (ix) −*x*−1/2, *y*−1/2, −*z*+1/2; (x) −*x*+1/2, *y*−1/2, −*z*+1/2; (xi) *x*−1/2, −*y*−1/2, *z*+1/2; (xii) −*x*+1/2, *y*+1/2, −*z*+1/2; (xiii) *x*+1/2, −*y*−1/2, *z*+1/2; (xiv) −*x*+1, −*y*, −*z*; (xv) *x*+1, *y*, *z*; (xvi) *x*−1/2, −*y*−1/2, *z*−1/2.
